# Use of Rat Mature Adipocyte-Derived Dedifferentiated Fat Cells as a Cell Source for Periodontal Tissue Regeneration

**DOI:** 10.3389/fphys.2016.00050

**Published:** 2016-02-23

**Authors:** Daisuke Akita, Koichiro Kano, Yoko Saito-Tamura, Takayuki Mashimo, Momoko Sato-Shionome, Niina Tsurumachi, Katsuyuki Yamanaka, Tadashi Kaneko, Taku Toriumi, Yoshinori Arai, Naoki Tsukimura, Taro Matsumoto, Tomohiko Ishigami, Keitaro Isokawa, Masaki Honda

**Affiliations:** ^1^Department of Partial Denture Prosthodontics, School of Dentistry, Nihon UniversityTokyo, Japan; ^2^Laboratory of Cell and Tissue Biology, College of Bioresource Science, Nihon UniversityFujisawa, Japan; ^3^Department of Orthodontics, School of Dentistry, Nihon UniversityTokyo, Japan; ^4^Department of Oral and Maxillofacial Surgery, Faculty of Medicine, Juntendo UniversityTokyo, Japan; ^5^Department of Pediatric Dentistry, School of Dentistry, Nihon UniversityTokyo, Japan; ^6^GC Corp. R&DTokyo, Japan; ^7^Department of Anatomy, School of Dentistry, Nihon UniversityTokyo, Japan; ^8^School of Dentistry, Nihon UniversityTokyo, Japan; ^9^Division of Cell Regeneration and Transplantation, Department of Functional Morphology, School of Medicine, Nihon UniversityTokyo, Japan; ^10^Department of Oral Anatomy, School of Dentistry, Aichi-Gakuin UniversityNagoya, Japan

**Keywords:** periodontal tissue regeneration, dedifferentiated fat cells (DFAT cells), PLGA scaffold, cell transplantation, periodontal fenestration defect

## Abstract

Lipid-free fibroblast-like cells, known as dedifferentiated fat (DFAT) cells, can be generated from mature adipocytes with a large single lipid droplet. DFAT cells can re-establish their active proliferation ability and can transdifferentiate into various cell types under appropriate culture conditions. The first objective of this study was to compare the multilineage differentiation potential of DFAT cells with that of adipose-derived stem cells (ASCs) on mesenchymal stem cells. We obtained DFAT cells and ASCs from inbred rats and found that rat DFAT cells possess higher osteogenic differentiation potential than rat ASCs. On the other hand, DFAT cells show similar adipogenic differentiation, and chondrogenic differentiation potential in comparison with ASCs. The second objective of this study was to assess the regenerative potential of DFAT cells combined with novel solid scaffolds composed of PLGA (Poly *d, l*-lactic-co-glycolic acid) on periodontal tissue, and to compare this with the regenerative potential of ASCs combined with PLGA scaffolds. Cultured DFAT cells and ASCs were seeded onto PLGA scaffolds (DFAT/PLGA and ASCs/PLGA) and transplanted into periodontal fenestration defects in rat mandible. Micro computed tomography analysis revealed a significantly higher amount of bone regeneration in the DFAT/PLGA group compared with that of ASCs/PLGA and PLGA-alone groups at 2, 3, and 5 weeks after transplantation. Similarly, histomorphometric analysis showed that DFAT/PLGA groups had significantly greater width of cementum, periodontal ligament and alveolar bone than ASCs/PLGA and PLGA-alone groups. In addition, transplanted fluorescent-labeled DFAT cells were observed in the periodontal ligament beside the newly formed bone and cementum. These findings suggest that DFAT cells have a greater potential for enhancing periodontal tissue regeneration than ASCs. Therefore, DFAT cells are a promising cell source for periodontium regeneration.

## Introduction

The use of adult mesenchymal stem cells (MSCs) seems to be ideal for practical periodontal regenerative medicine, because they are not subject to the restrictions such as embryonic stem cells (ESCs) or induced pluripotent stem cells (Prockop, 1997; Pittenger et al., 1999; Zuk et al., 2001; Reyes et al., 2002; Safford et al., 2002; Akita et al., 2014). ASCs may be a promising cell source for periodontal-tissue regeneration (Tobita et al., 2008; Tobita and Mizuno, 2013; Akita et al., 2014). The use of ASCs has several advantages over bone marrow-derived or dental tissue-derived MSCs such as dental follicle (Honda et al., 2007, 2010, 2011), dental pulp (Aurrekoetxea et al., 2015; Sato et al., 2015), and periodontal ligament (Saito et al., 2013). Adipose tissue contains large numbers of stromal cells and is available in larger quantities than bone marrow and dental tissue (Fraser et al., 2006). Furthermore, the method for obtaining adipose tissue is relatively easy and is less invasive than that for bone marrow, and adipose tissue can be obtained from patients without teeth. The utility of ASCs for periodontal tissue regeneration has been demonstrated in rat and canine models in several studies, but successful use of human ASCs for such purpose has not been described yet (Tobita et al., 2008; Tobita and Mizuno, 2013; Akita et al., 2014).

Adipose tissue is connective tissue composed mostly of adipocytes surrounded by fibroblasts. Mature adipocytes with a large single lipid droplet are generally considered to be terminally differentiated cells which have lost their proliferative ability. However, by using a ceiling culture methods based on buoyancy, lipid-free fibroblast-like cells, known as dedifferentiated fat (DFAT) cells, can be separated from mature adipocytes in our previous studies (Yagi et al., 2004; Nobusue et al., 2008; Nobusue and Kano, 2010). DFAT cells can re-establish their active proliferation ability and can differentiate into various cell types under appropriate culture conditions (Matsumoto et al., 2008; Kono et al., 2014). DFAT cells may have the potential to contribute to dental tissue regeneration (Kaku et al., 2015).

A three-dimensional (3D) biodegradable scaffold is likely to be necessary for the delivery of stem cells or progenitor cells to periodontal defect, both to preserve space for the formation of new periodontal tissue and to provide initial support for the growing transplanted cells (Wang et al., 2005). Since the most extensively used synthetic polymers in tissue engineering studies are poly(glycolic acid), poly(lactic acid), and their copolymers (e.g., Poly *d, l*-lactic-co-glycolic acid; PLGA), we previously examined the *in vivo* performance of solid PLGA scaffolds seeded with ASCs (Akita et al., 2014). Solid PLGA scaffolds have large fully interconnected pores and substantially higher compressive strength than sponge-like PLGA-based scaffolds. Recently, the possibility of using DFAT cells to promote periodontal tissue regeneration was raised by researchers who seeded an atelocollagen sponge-like scaffold with DFAT cells (Sugawara and Sato, 2014). An advantage of the higher compressive strength of solid PLGA scaffolds is that they typically offers higher primary stability than natural scaffolds such as those composed of atelocollagen. Our results showed that the PLGA scaffolds maintained their structural integrity for 5 weeks when used for *in vivo* transplants (Akita et al., 2014). We concluded that these solid PLGA scaffolds are useful for regeneration of periodontium.

To date, no studies evaluating DFAT cells combined with solid PLGA scaffolds for periodontal tissue regeneration have been published. We first compared the characteristics of rat DFAT cells with those of rat ASCs—including proliferative and multipotent differentiation potential. We then evaluated the *in vivo* potential for periodontal tissue regeneration of rat DFAT cells combined with solid PLGA scaffolds in periodontal fenestration defects created in mandibular alveolar bone, and compared the performance of rat DFAT cells in this context with that of ASCs.

## Materials and methods

All animal experiments were reviewed and approved by the Animal Research and Care Committee at the Nihon University School of Dentistry (AP10D014 and AP15D006).

### Isolation of rat DFAT cells and ASCs

**Figure 1:**
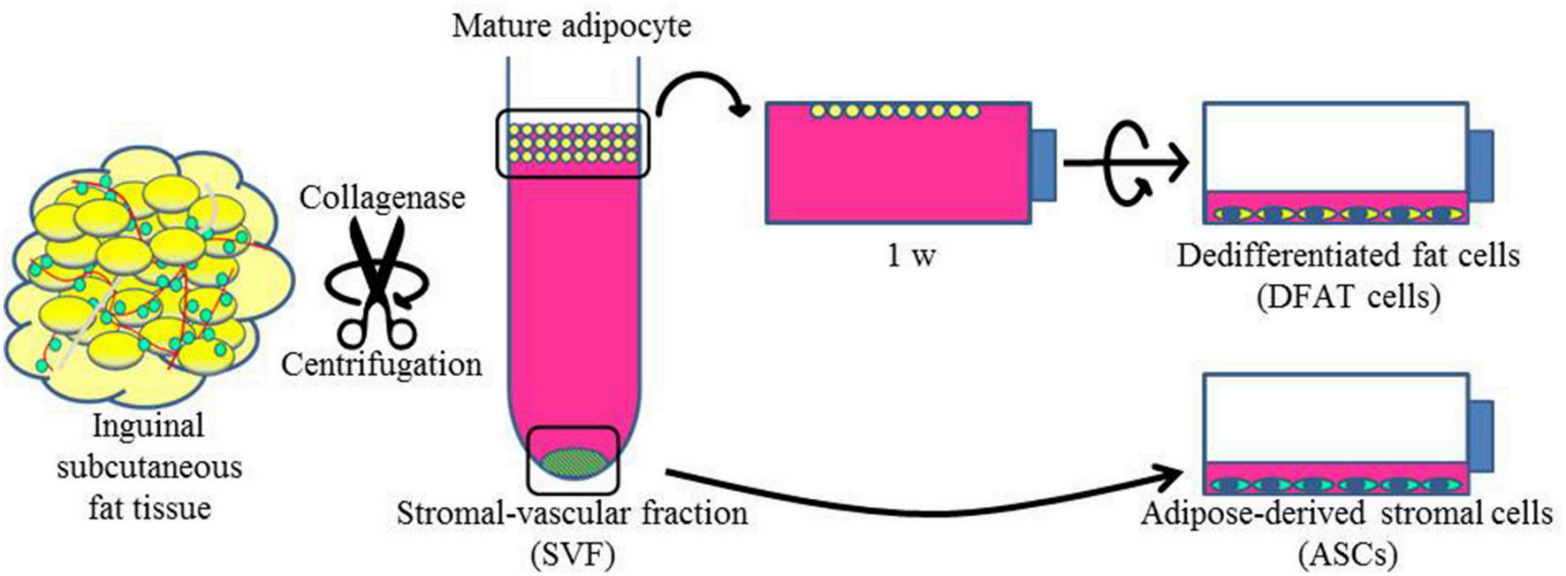
**Isolation of DFAT cells and ASCs**. The upper section shows the method used to isolate DFAT cells from floating unilocular adipocytes. The floating cells were attached to the upper surface of the flasks and then DFAT cells emerged by asymmetrical division of floating cells for 1 week. The lower section shows the method used to isolate ASCs. After centrifugation, the SVF fraction was separated by sedimentation from floating cells and the SVF fraction was cultured for isolation of ASCs.

To isolate DFAT cells and ASCs, 9-week-old male F344 rats (*n* = 5, body weight 190 ± 10 g) were purchased from CLEA Japan, Inc. (Tokyo, Japan). Isolation of DFAT cells from mature adipocytes was done with a modified version of a method that has been described previously (Matsumoto et al., 2008). Briefly, ~1 g of inguinal subcutaneous fat tissue was washed extensively with phosphate-buffered saline (PBS; Wako, Osaka, Japan) and minced and digested in 0.1% (w/v) collagenase solution (C6885; Sigma-Aldrich, St. Louis, MO) at 37°C for 60 min with gentle agitation. After filtration and centrifugation at 135 g for 3 min, the floating primary mature adipocytes in the top layer were collected. After three washes with PBS, cells (5 × 10^4^) were placed in 12.5 cm^2^ culture flasks (BD Falcon, England) filled completely with Dulbecco's modified Eagle's medium (DMEM; Sigma-Aldrich, UK) and supplemented with 20% fetal bovine serum (FBS; Nichirei Bioscience Inc., Tokyo, Japan), and were incubated at 37°C in 5% CO_2_. Mature adipocytes floated up and adhered to the top inner surface (ceiling surface) of the flasks. After about a week, the medium was removed and changed into DMEM supplemented with 20% FBS, and the flasks were inverted so that the cells were on the bottom (Figure 1). The medium was changed every 4 days until the cells reached confluence.

Cultured ASCs were prepared as described previously (Tobita et al., 2008; Tobita and Mizuno, 2013; Akita et al., 2014). Briefly, the stromal vascular fraction (SVF) was isolated as the pellet fraction from collagenase-digested adipose tissue by centrifugation at 180 g for 5 min after collecting of the floating uppermost layer as described above. The remaining cells were plated in DMEM supplemented with 20% FBS and 1% antibiotics as a growth medium. The cells were referred to as SVF cells, and were cultured at 37°C in a humidified atmosphere containing 5% CO_2_ (Figure 1). In the comparison experiments, second and third passage ASCs and DFAT cells from the same samples were used. All animal experiments were reviewed and approved by the Animal Research and Care Committee at the Nihon University School of Dentistry (AP10D014 and AP15D006).

### Adipogenic, osteoblastic, and chondrocytic differentiation *in vitro*

Differentiation assays for adipocytes, osteoblasts, and chondrocytes were performed as previously described (Matsumoto et al., 2008; Mikami et al., 2011; Saito et al., 2013; Akita et al., 2014; Sato et al., 2015). The three cell lines obtained from three rats were used in those studies.

For adipogenic differentiation, the cells were plated in 6-well plates at a density of 5 × 10^4^ cells and grown to confluency with growth medium. The cells were then incubated for 21 days in DMEM containing 10% FBS, 1 mM dexamethasone (Sigma-Aldrich), 0.5 mM isobutylmethylxanthine (Sigma-Aldrich), and 1× insulin-transferrin-selenium-A (ITS; GIBCO, Grand Island, NY) as an adipogenic induction medium, and lipid vacuole formation was examined by Oil red O staining (Wako Pure Chemical Industries, Osaka, Japan). To evaluate the adipogenic differentiation ability, Oil red O-positive cells were count under the phase contrast microscope.

For osteogenic differentiation, cells were plated in 6-well plates at a density of 5 × 10^4^ cells and grown to confluency with growth medium. The growth medium was replaced with osteogenic induction medium including DMEM containing 10% FBS, 100 nM dexamethasone, 10 mM glycerol 2-phosphate disodium salt hydrate (Sigma-Aldrich), and 50 mM L-ascorbic acid phosphate magnesium salt *n*-hydrate (Wako Pure Chemical Industries). The cells were cultured with the osteogenic induction medium for 21 days and stained with alkaline phosphatase (ALP) activity using nitro-blue tetrazolium plus 5-bromo-4-chloro-3′-indolyphosphate (NBT/BCIP) ready-to-use tablets, pH 9.5 (Roche Diagnostics, Pentzberg, Germany). To detect calcium deposition, cells were incubated in 1% Alizarin red S (Sigma-Aldrich). Ca^2+^ evaluated was also determined by measuring Ca^2+^ uptake using the Calcium *E*-test (Wako Pure Chemical Industries).

For chondrogenic differentiation, cell pellets were collected in centrifuge tube (2.5 × 10^4^ cells/tube) with growth medium. After 2 days in culture, the growth medium was replaced with chondrogenic induction medium including DMEM containing 1% FBS, 50 μM L-ascorbic acid phosphate magnesium salt n-hydrate, 40 μg/mL proline (Sigma-Aldrich), 100 μg/mL pyruvate (Sigma-Aldrich), 10 ng/mL recombinant human TGF-β3 (R&D Systems, Inc., Minneapolis, MN, USA), and 1 × ITS for 21 days. The microscopic structure of cells was examined after cell pellets were fixed for 10 min with 10% neutral buffered paraformaldehyde, dehydrated in a graded series of ethanol solutions, embedded in paraffin, and cut into 7 μm sections. After deparaffinization, sections were stained with Alcian blue (0.1 N, pH 1.0). Each test was conducted three times and results are presented as the mean ± standard deviation.

### Preparation of cell-scaffold complex

The PLGA-based solid scaffolds (LA:GA = 75:25, Mw. 25 kDa; PLGA scaffold, GC Dental Product Co. Ltd.), with a porosity of 80%, were prepared as previously described (Akita et al., 2014; Yamanaka et al., 2015). Briefly, PLGA scaffolds were resized to ~2 × 3 × 1 mm^3^ and soaked in 70% ethanol for 30 min to improve wetting, and then a vacuum pump was used to remove air bubbles. The scaffolds were then washed three times (30 min each) in PBS to remove residual ethanol, and 100 μL aliquots of the cell suspension (1 × 10^7^ cells/mL, 1 × 10^6^ cells/scaffold) were seeded onto the tops of prewetted PLGA scaffolds and left undisturbed in an incubator for 1 h. As a negative control, empty PLGA scaffolds were prepared.

### Cell transplantation

**Figure 2:**
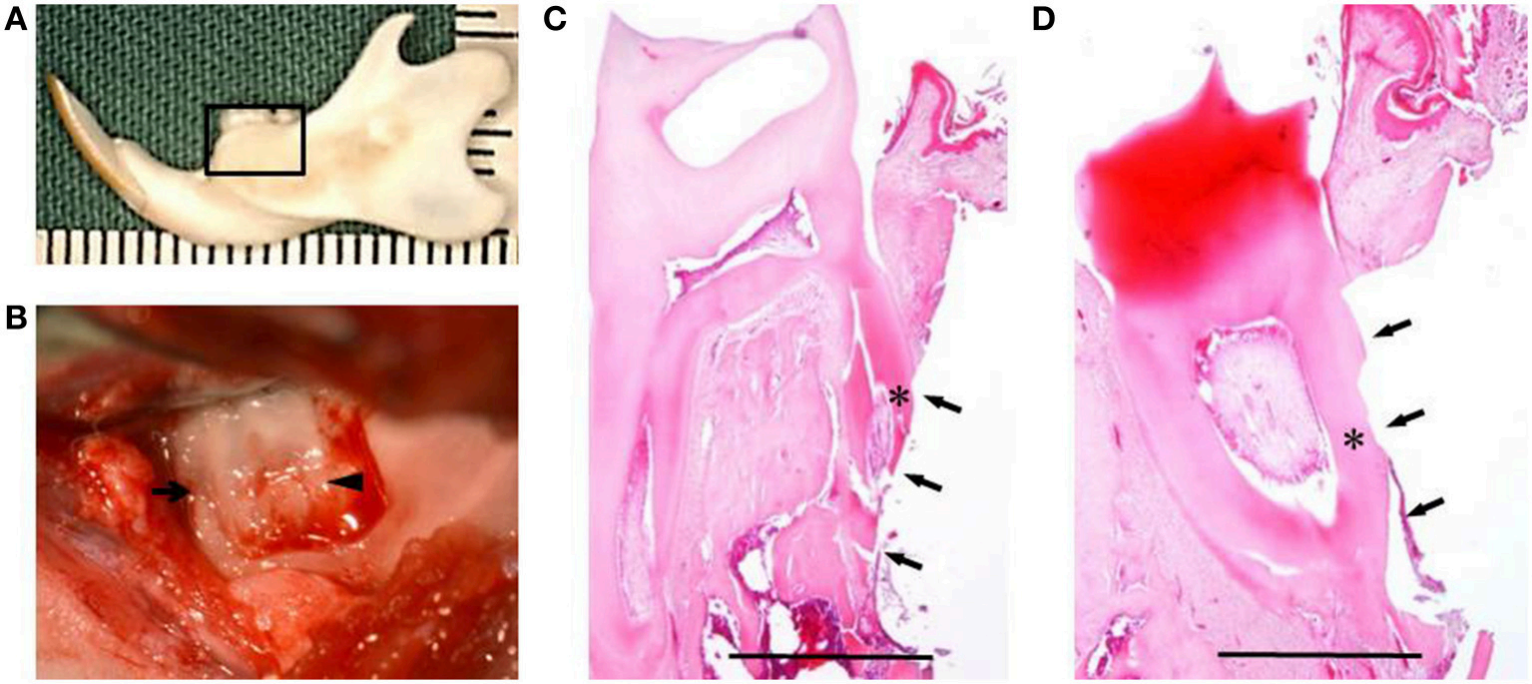
**Creation of the periodontal fenestration defect. (A)** Rat mandibular frame specimen. The black flame indicates the site of the surgically created periodontal fenestration defect site. **(B)** Macroscopic view of a surgically created periodontal defect on the buccal surface of the mandibular first molar. The arrow indicates the first molar mesial root and the arrowhead indicates the first molar distal root. **(C)** H&E staining showing the surgically exposed central root of the first mandibular molar. The defect includes alveolar bone, periodontal ligament, and cementum. The arrows indicate the defect site and the asterisk indicates the central root of the first molar on the buccal side. Scale bar = 1000 μm. **(D)** H&E staining showing surgically exposed distal root of the first mandibular molar. The arrows indicate the defect site and the asterisk indicates the distal root of the first molar on the buccal side. Scale bar = 1000 μm.

Fifteen healthy male F344 rats (10-weeks-old; 200 ± 10 g) were used for *in vivo* experiment. Under inhalational device of Isoflurane (KN-1071 Marcobit-E; Natsume Seisakusho Co. Ltd., Tokyo, Japan), the rat periodontal fenestration defect model was prepared as previously described (King et al., 1997; Yang et al., 2010; Han et al., 2013; Akita et al., 2014). A skin incision was made along the inferior border of the left mandible, then the masseter muscle and the periosteum covering the buccal surface of the mandible were elevated as a flap. The alveolar bone overlying the mandibular first and second molar roots was removed with a dental inverted bur. The size of the periodontal defect was ~2 × 3 × 1 mm in height, width, and depth, respectively, with the anterior margin mesial to the distal root of the first molar and the posterior margin just distal to the second molar. The coronal margin was ~1.5 mm apical to the crest of the alveolar bone and the inferior margin was ~2.5 mm apical to the alveolar crest (Figures 2A,B). The exposed roots of the first and second molars were denuded and cementum was completely removed together with the supporting bone, which occasionally resulted in penetration into the root dentin (Figures 2C,D). PLGA scaffolds with DFAT (DFAT/PLGA) or ASCs (ASCs/PLGA) were placed in the defects and covered with membrane (GC membrane; GC Dental Product Co. Ltd.). The unilateral mandibular first molar in each of the 15 rats were selected for analysis and the defects were randomly assigned to the following three treatment groups: (1) PLGA scaffolds covered with GC membrane (GC membrane; GC Dental Product Co. Ltd.) without cells (PLGA-alone group) (*n* = 5), (2) ASCs-loaded PLGA scaffolds covered with membrane (ASCs/PLGA group) (*n* = 5), and (3) DFAT cells-loaded PLGA scaffolds covered with membrane (DFAT/PLGA group) (*n* = 5). The right side of the mandibular was not operated to maintain oral function. After the masseter muscle was repositioned, the skin incision was closed to ensure healing by primary intention.

### *In vivo* micro computed tomography (CT) imaging and analysis

*In vivo* x-ray micro computed tomography (R_mCT; Rigaku Corporation, Tokyo, Japan) was used as previously described (Akita et al., 2014). The exposure parameters were 17 s, 90 kV, and 100 μA. The isotropic voxel size was 30 μm.

Briefly, hard tissue regeneration images were obtained from each rats immediately after surgery and each week until 5 weeks after surgery. The images were constructed into 3D images using i-View (J. Morita Co., Kyoto, Japan). Bone volume was measured in the regions of interest (ROIs) from voxel images using bone volume-measuring software 3 by 4 viewer 2011 (Kitasenjyu Radist Dental Clinic I-View Image Center, Tokyo, Japan). The ROI size was 4 × 3 × 1.5 mm, which covered the surgically created periodontal tissue defect. The bone volume in the ROI was measured immediately after surgery and each week until 5 weeks after surgery. The increase in bone volume in individual rats was then calculated by subtracting the bone volume on day 0 from each of the subsequent values. The increase in bone was considered to be defect re-ossification.

### Histological and histometric analysis

Five weeks after surgery, the harvested specimens were fixed in 10% neutral buffered paraformaldehyde for 24 h, decalcified in 10% ethylenediaminetetraacetic acid (EDTA) for 5 weeks, dehydrated through a graded series of ethanol solutions, and then embedded in paraffin. For the rat specimens, frontal plane sections (7 μm thick) were prepared with a microtome (Leica RM2165, Nussloch, Germany) and the paraffin sections of the first molar central and distal roots were stained with hematoxylin and eosin (H&E). To observe the periodontal ligament fibers and Sharpey's fibers, the paraffin sections were stained with Azan and Picrosirius-red.

For the quantitative analysis, five H&E-stained sections per specimen were selected and the thickness of newly formed cementum and periodontal ligament in the first molar central and distal roots were measured by light microscopy (ECLIPSE LV100POL, Nikon, Tokyo, Japan).

After decalcification, the sections were stained as follows: dewaxing and rehydration; immersion in 0.1% Picrosirius-red solution (Sirius red 0.1 g dissolved into 100 mL of saturated picric acid solution) for 1 h of staining; rinsing with water for 5 min; re-staining with Harris hematoxylin for 5 min, dehydration with a gradient series of ethanol solutions; treatment with a xylene solution; and sealing with a rubber mount. The amount, distribution, and morphology of each different type of collagen were assessed by polarized light microscopy.

### Localization of fluorescent-labeled DFAT cells

Four healthy male F344 rats (10-weeks-old; 200±10 g) were used for this experiment. Cells harvested with 0.25% trypsin-EDTA, resuspended at 1 × 10^6^ cells/ml in DMEM, and labeled with a fluorescent dye—chloromethylbenzamido 1,1′ -dioctadecyl- 3,3,3′,3′ tetramethylindocarbocyanine (DiI; Vybrant®, V22885, Life Technologies, Eugene, OR, USA). Fluorescent lipophilic tracer was added at 5 μL/mL in DMEM. After incubation for 20 min at 37°C with 5% humidified CO_2_, the cells were centrifuged at 180 g for 5 min and washed twice with PBS. To prepare cryosections, fixed, and dehydrated specimens after 5 weeks fluorescent-labeled cell transplantation were embedded immediately in OCT compound (Sakura Finetechnical Co. Ltd., Tokyo) and frozen in liquid nitrogen. After cryosections were incubated for 30 min with a fluorescent 4′,6-diamidino-2-phenylindole solution (DAPI; Sigma-Aldrich), fluorescence microscopy (Biozero BZ-8000, Keyence, Osaka, Japan) was used to investigate the survival and localization of transplanted cells.

### Statistical analysis

Data was expressed as the mean and standard deviation (SD) for each group. Statistical analysis was performed using ExcelStatistical File software (ystat2008.xls; Igakutosyosyuppan, Tokyo, Japan). The Mann–Whitney *U*-test was used for intergroup comparisons. *P* < 0.05 was considered statistically significant.

## Results

### Characterization of rat DFAT cells

**Figure 3:**
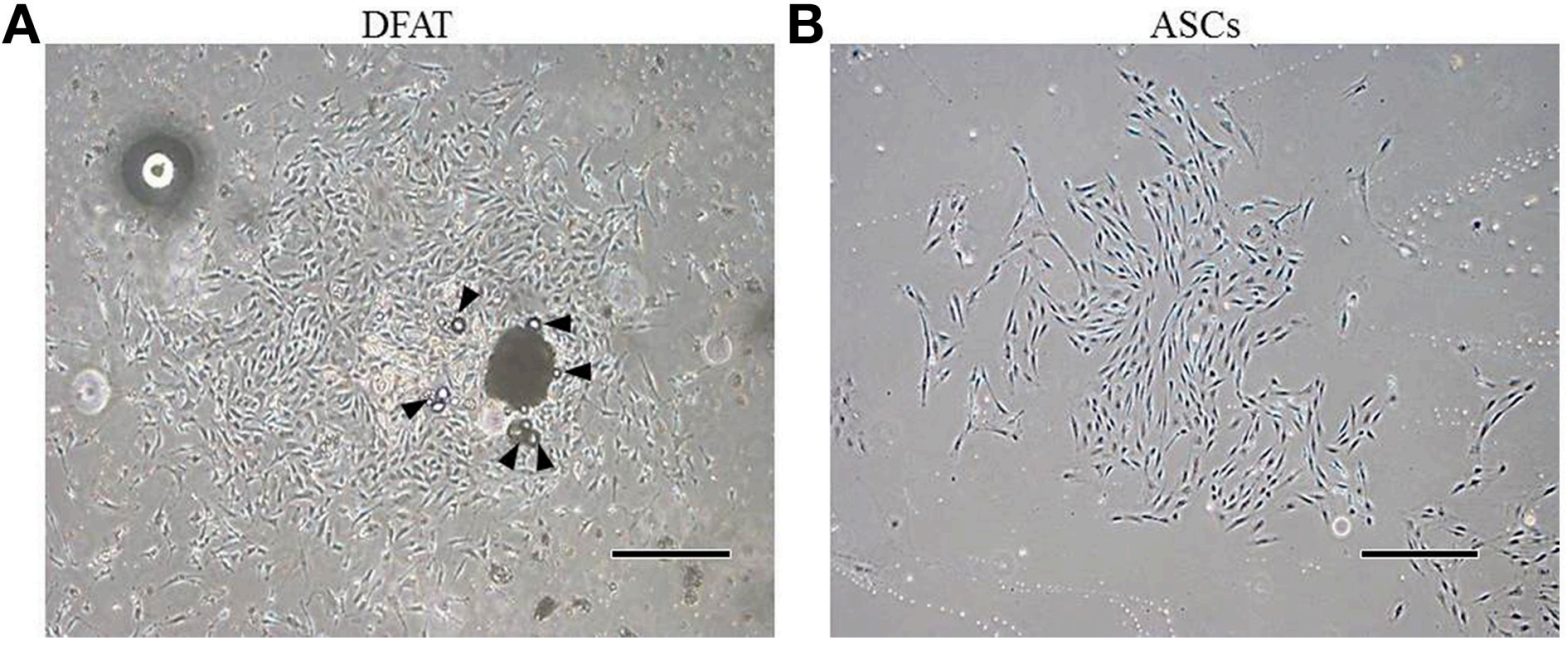
**Morphology of primary cultured DFAT cells and ASCs. (A)** When the flasks had been inverted for 7 days, DFAT cells grew to form a colony. Some DFAT cells in the colony still have a lipid droplet (arrowheads). Scale bar = 500 μm. **(B)** Cultured ASCs are more spindle-shaped than DFAT cells. Scale bar = 500 μm.

After ceiling culture, the adhered mature adipocytes divided asymmetrically and generated cuboidal cells with scanty cytoplasm as described previously (Matsumoto et al., 2008). The cuboidal cells formed colonies on day 7 (Figure 3A). We also successfully prepared ASCs from SVF fractions; these cells also expanded and formed colonies on day 7 as described in our previous reports (Figure 3B) (Akita et al., 2014). The morphology of the ASCs was slightly different from that of the cells derived from mature adipocytes. The ASCs have more cytoplasm than the DFAT cells.

**Figure 4:**
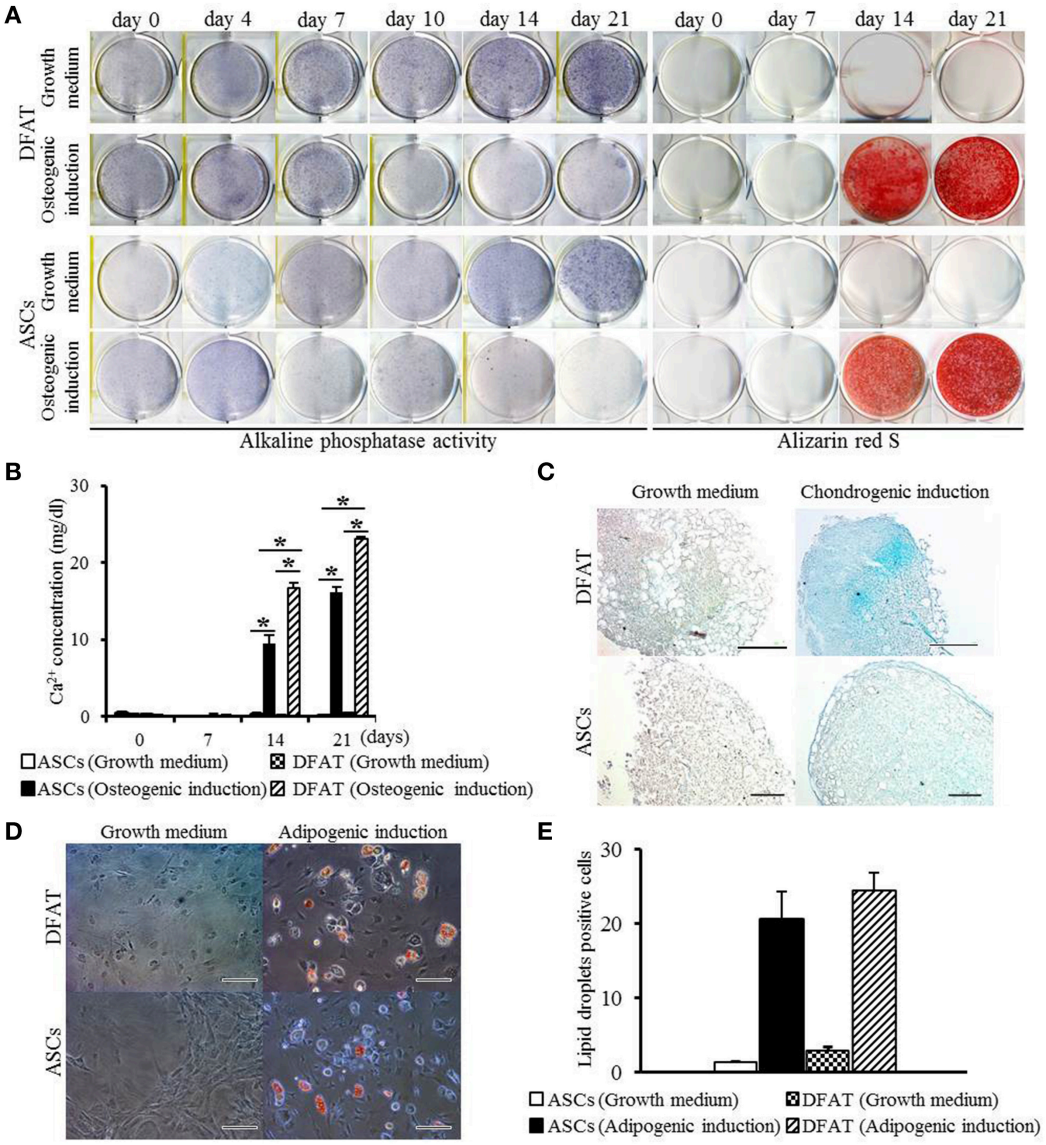
**Multilineage potential of DFAT cells and ASCs. (A)** ALP activity and Alizarin red staining, examined to detect osteogenic potential of DFAT cells, and ASCs. **(B)** Calcium concentration determined by quantitative colorimetric assay. Each bar represents the mean ± SD (*n* = 12); ^*^P < 0.05. **(C)** Cells stained for Alcian blue to determine chondrogenic differentiation. Scale bar = 100 μm. **(D)** Adipogenic differentiation evaluated by staining with Oil red O. **(E)** Oil red O-positive lipid droplets were measured in DFAT cells and ASCs under growth and adipogenic induction media. There was no significant difference between DFAT cells and ASCs on the cell number stained by Oil red O.

Next, differentiation potential was analyzed. Patterns of increase in ALP activity were detected in both cell populations with or without differentiation-inducing medium. Since the extent of the formation of Alizarin Red-positive mineralized nodules in DFAT cells was slightly higher than that observed in ASCs on day 14 (Figure 4A), calcium accumulation was measured. Calcium accumulation in osteogenic induction medium was significantly higher in DFAT cells than in ASCs at day 14 and 21 (Figure 4B). Newly synthesized glycosaminoglycan, as determined by Alcian-blue positive staining, was observed in both cell populations when subjected to conditions that favor chondrogenic differentiation (Figure 4C). DFAT cells showed Alcian-blue positive areas in the cell pellets in chondrogenic induction medium compared with that of ASCs. The accumulation of lipid vacuoles as determined by positive Oil red O staining was observed in the cytoplasm in both cell populations when subjected to conditions that favor adipogenic differentiation (Figure 4D). The number of Oil red O-positive cells in the DFAT cell population was similar to that in the ASC population (Figure 4E).

### Hard tissue formation

**Figure 5:**
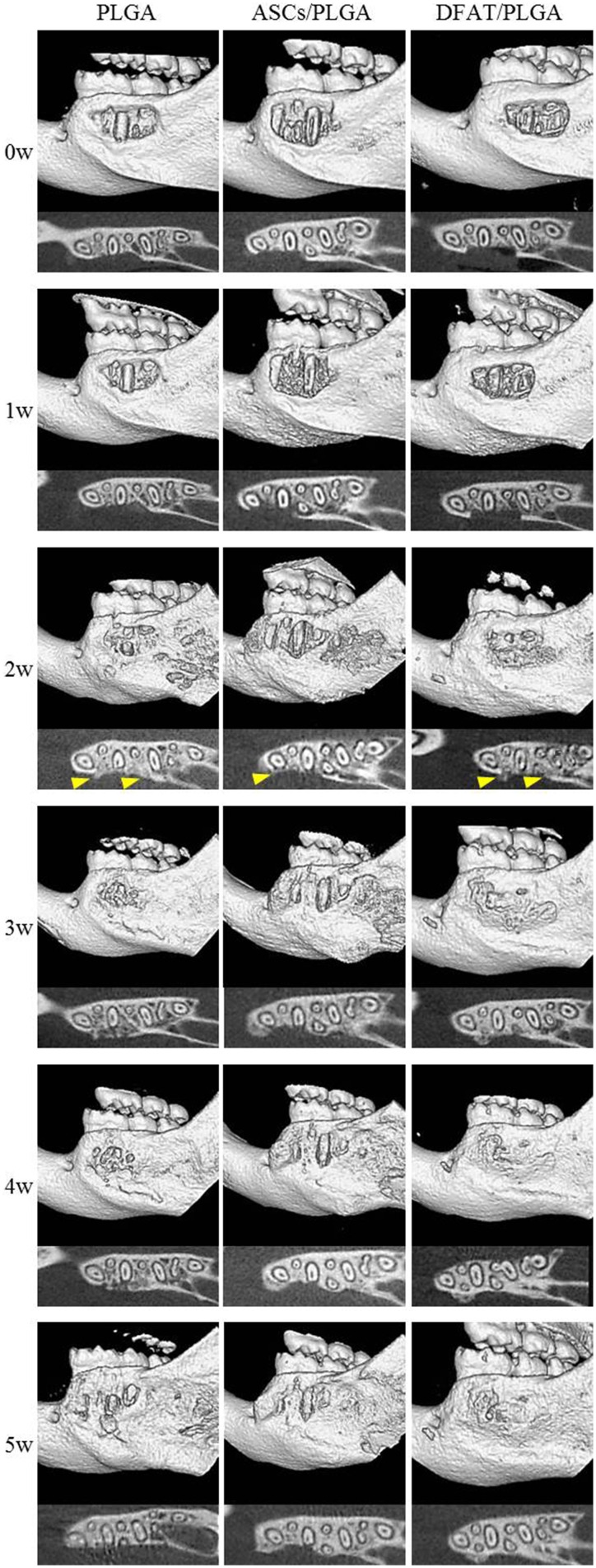
**Micro-CT images of regenerative process in the periodontal fenestration defect**. Micro-CT images were obtained from the identical rats immediately after transplantation until 5 weeks after transplantation. The upper section of each image shows reconstructed micro-CT images. The lower section shows the horizontal plane section of the mandible. Hard tissue is visible (distinct from native bone structure) at 2 weeks (arrowheads).

Furcation periodontal tissue defects were artificially prepared and transplanted with PLGA-alone, ASCs/PLGA, or DFAT/PLGA scaffolds. No severe inflammation or swelling was observed in any examined sites throughout the experimental period. To evaluate hard tissue formation in the alveolar bone areas, reconstituted micro computed tomography (micro-CT) images and horizontal sections were prepared by micro-CT until week 5 because the artificially created defect area was almost filled with newly formed hard tissue by week 5 in the DFAT/PLGA scaffold group (Figure 5).

**Figure 6:**
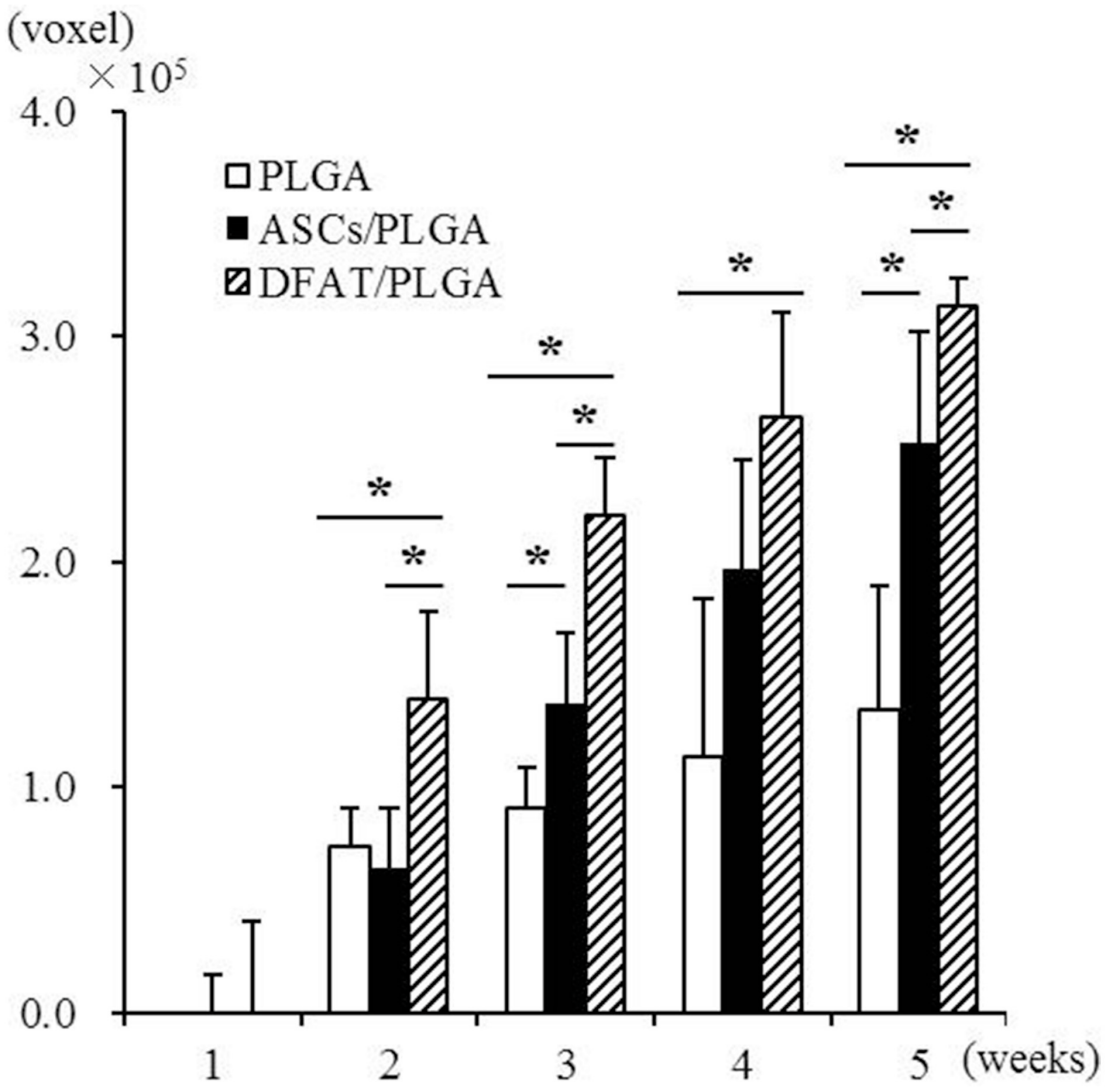
**Quantification of newly formed hard tissue regeneration using bone volume measuring software and the data from micro-CT analysis**. A significant difference was observed between the DFAT/PLGA and ASCs/PLGA groups at weeks 2, 3, and 5 (*n* = 12, ^*^ *P* < 0.05).

Newly formed hard tissue was clearly visible at the alveolar bone defect sites in all groups at week 2. Interestingly, cortical bone-like tissue was observed at the outer layer of the defect sites at 4 weeks in the DFAT/PLGA scaffold, but not the ASCs/PLGA and PLGA-alone scaffolds by the horizontal images. At 5 weeks, the newly formed hard tissue was completely connected with the native cortical bone at both mesial and distal side in the DFAT/PLGA group. The translucent image between dental root and the newly formed hard tissue was clearly visible in all the samples by week 5. Since the DFAT/PLGA scaffolds showed large amounts of newly formed hard tissue compared with the ASCs/PLGA scaffolds, we calculated the quantity of new hard tissue formation. The hard tissue volume in DFAT/PLGA scaffolds was significantly higher than that in ASCs/PLGA scaffolds at 2, 3, and 5 weeks (Figure 6) (*P* < 0.05).

### Transplantation of DFAT/PLGA enhanced periodontal tissue regeneration

**Figure 7:**
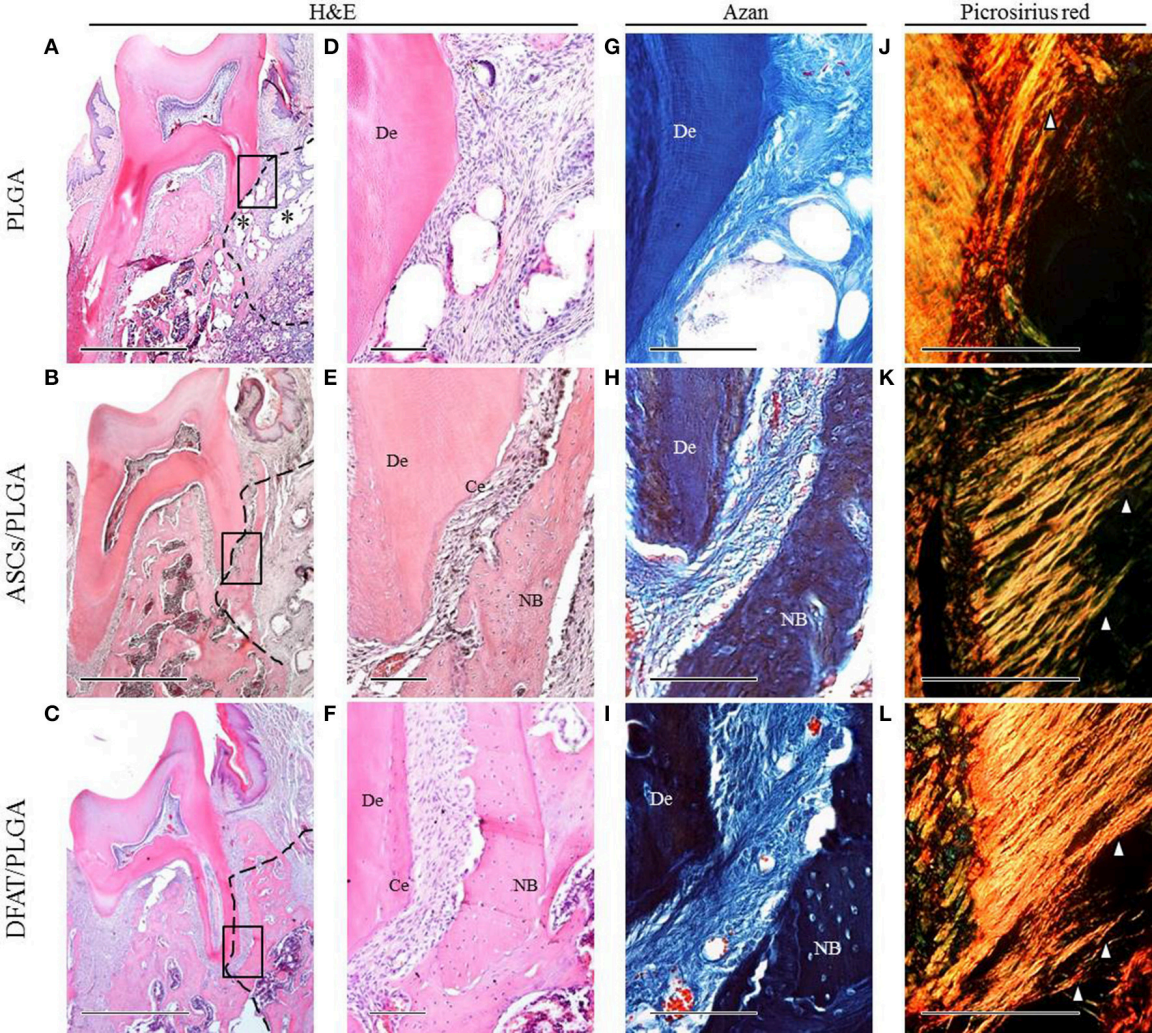
**Representative histological observation of frontal plane section in central root of first molar. (A–C)** Overview of H&E staining. No alveolar bone formation was observed in the PLGA-alone group. Asterisk indicate scaffold remnant. Alveolar bone formation was observed in the ASCs/PLGA and DFAT/PLGA groups. The dotted line indicates defect site. Scale bar = 1000 μm. **(D–F)** Higher magnification of the framed areas in **(A–C)**, respectively. No cementum formation was observed in the PLGA-alone group and newly formed cementum was observed in the ASCs/PLGA and DFAT/PLGA groups. Scale bar = 100 μm. **(G–I)** Higher magnification of the framed area in **(A–C)**, respectively, with Azan staining to observe the periodontal ligament fibers. Scale bar = 100 μm. **(J–L)** Enlarged images of the area equivalent to **(G–I)**, stained with Picrosirius-red to show Sharpey's fibers. Arrowheads indicate newly formed collagen fibers. Scale bar = 100 μm. NB, newly formed bone; Ce, newly formed cementum; De, root dentin.

**Figure 8:**
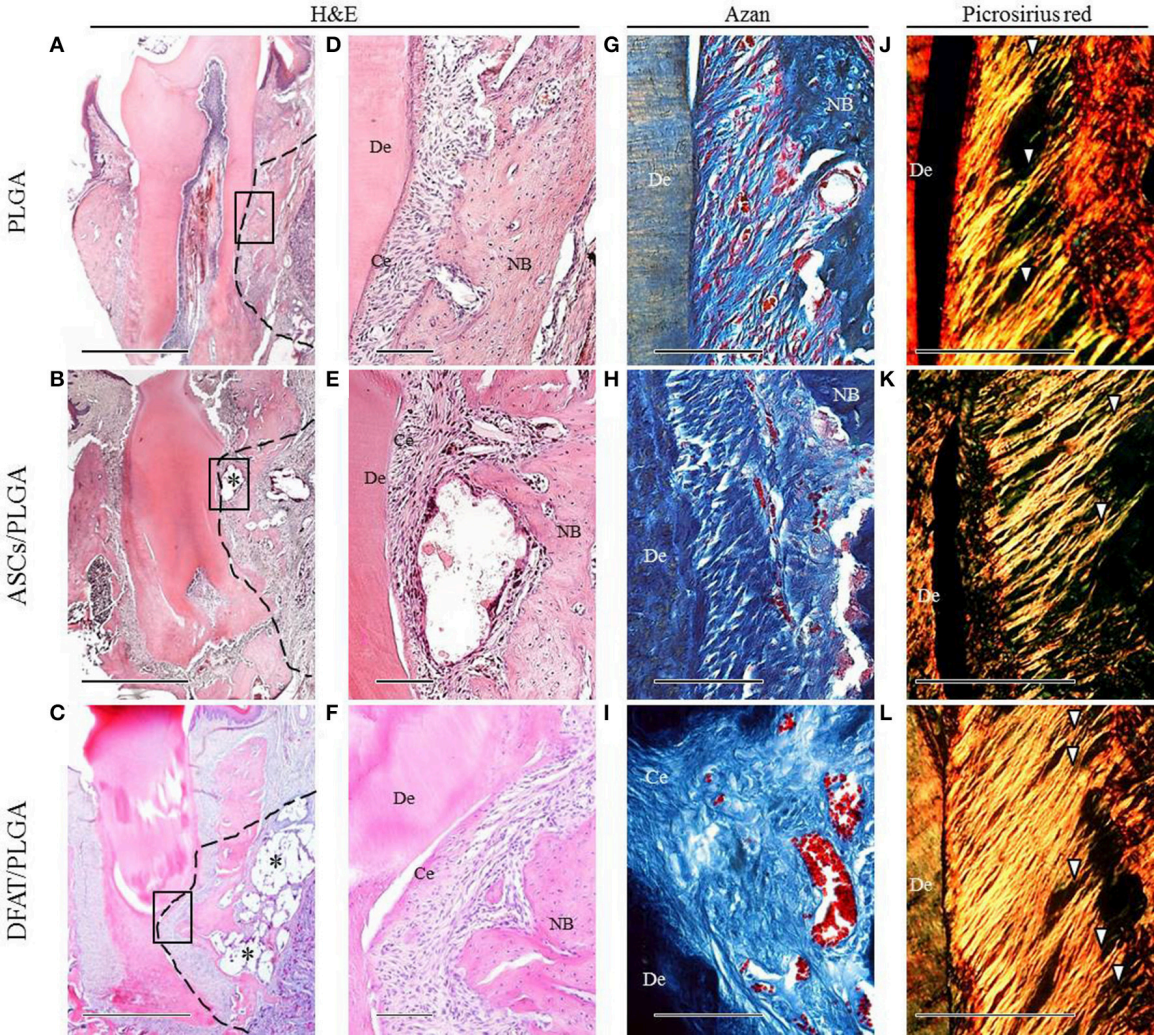
**Representative histological observation of frontal plane section in distal root of first molar. (A–C)** Overview of H&E staining showing significant bone formation in DFAT/PLGA and ASCs/PLGA groups. Asterisks indicate scaffold remnants. The dotted line indicates defect sites. Scale bar = 1000 μm. **(D–F)** Higher magnification of the framed area in **(A–C)**, respectively. Newly formed abundant cementum was observed in the DFAT/PLGA group. Scale bar = 100 μm. **(G–I)** Azan staining at higher magnification showed collagen bundles in the PLGA-alone group. Collagen bundles of more than 100 μm width were observed in the ASCs/PLGA and DFAT/PLGA groups. Scale bar = 100 μm. **(J–L)** Enlarged images of the area equivalent to **(G–I)**, stained with Picrosirius-red staining to show periodontal ligament fibers. Arrowheads indicate newly formed collagen fibers. Scale bar = 100 μm. NB, newly formed bone; Ce, newly formed cementum; De, root dentin.

By week 5, the created defect sites were easily identifiable by H&E in the PLGA-alone group because the remnant of the PLGA scaffold was clearly visible (Figures 7A,D, 8A,D). At low magnification, formation of mineralized tissues such as bone and cementum was evident in the DFAT/PLGA and ASCs/DFAT groups at 5 weeks (Figures 7B,C), but not in the PLGA-alone group (Figure 7A). The newly formed bone structures within the defect were coalesced with surrounding native alveolar bone (Figures 7B,C, 8A–C). The presence of osteoblasts lining the organic matrix and osteocytes trapped in the lacunae of newly formed bone structures was detected in the DFAT/PLGA and ASCs/PLGA groups (Figures 7E,F, 8E,F). Vascularization was observed in all samples (Figures 7G–I, 8G–I).

Periodontal ligament organization was recognized in the cementum-ligament-alveolar bone complex in the DFAT/PLGA and ASCs/PLGA groups (Figures 7H,I, 8G–I). The orientation of the ligament fibers, visualized by Picrosirius-red staining, resembled the oblique fibers in the functional native periodontal ligament tissue (Figures 7K,L, 8K,L). In the PLGA-alone group, sparse, and disorganized fibers were observed between dentin and scaffold because there was no cementum or nearby bone formation in the alveolar bone defect (Figures 7J, 8J).

**Figure 9:**
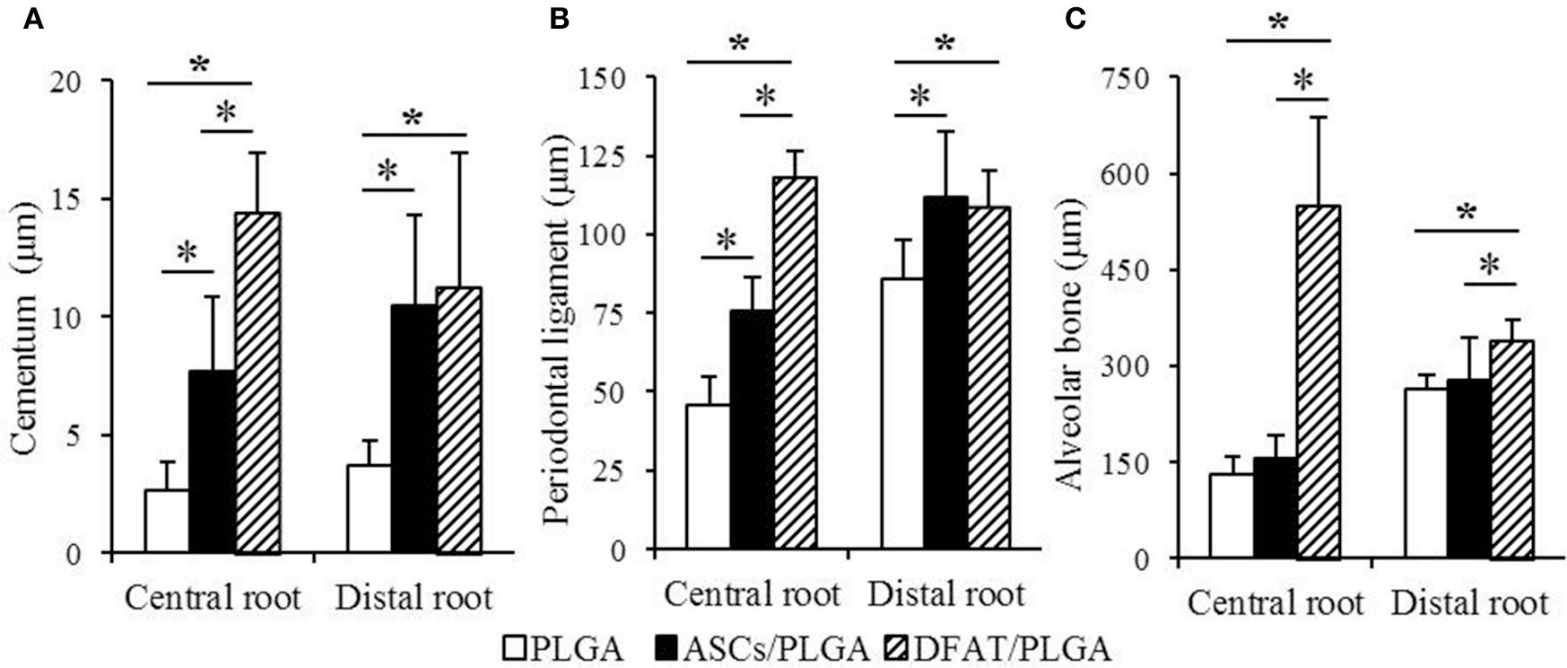
**Histometric analysis of newly formed cementum thickness, periodontal ligament width, and alveolar bone width. (A)** In the central root of the first molar, the cementum in the DFAT/PLGA group was significantly thicker than that in the ASCs/PLGA and PLGA-alone groups. In the distal root of the first molar, there were no significant differences between DFAT/PLGA and ASCs/PLGA. **(B)** In the central root of first molar, the periodontal ligament of the DFAT/PLGA group was significantly wider than that of the ASCs/PLGA and PLGA-alone groups. In the distal root of first molar, there were no significant differences between DFAT/PLGA and ASCs/PLGA groups. **(C)** In the central root and distal root of the first molar, the alveolar bone in the DFAT/PLGA group was significantly thicker than in the ASCs/PLGA and PLGA-alone groups (*n* = 12, ^*^P < 0.05).

The results of the histometric analysis are summarized in Figure 9. The newly formed cementum on the central root in the DFAT/PLGA group was significantly thicker than that in the ASCs/PLGA and PLGA-alone groups (*P* < 0.05) (Figure 9A). Furthermore, the thickness of periodontal ligament regenerated in the tissue defects at the part of the central root in the DFAT/PLGA group was also significantly larger than in the ASCs/PLGA and PLGA-alone groups (*P* < 0.05) (Figure 9B). Finally, the newly formed alveolar bone at the part of central root in the DFAT/PLGA group was significantly thicker than that in the ASCs/PLGA and PLGA-alone groups.

### Localization of transplanted DFAT cells in the periodontal tissue defects

**Figure 10:**
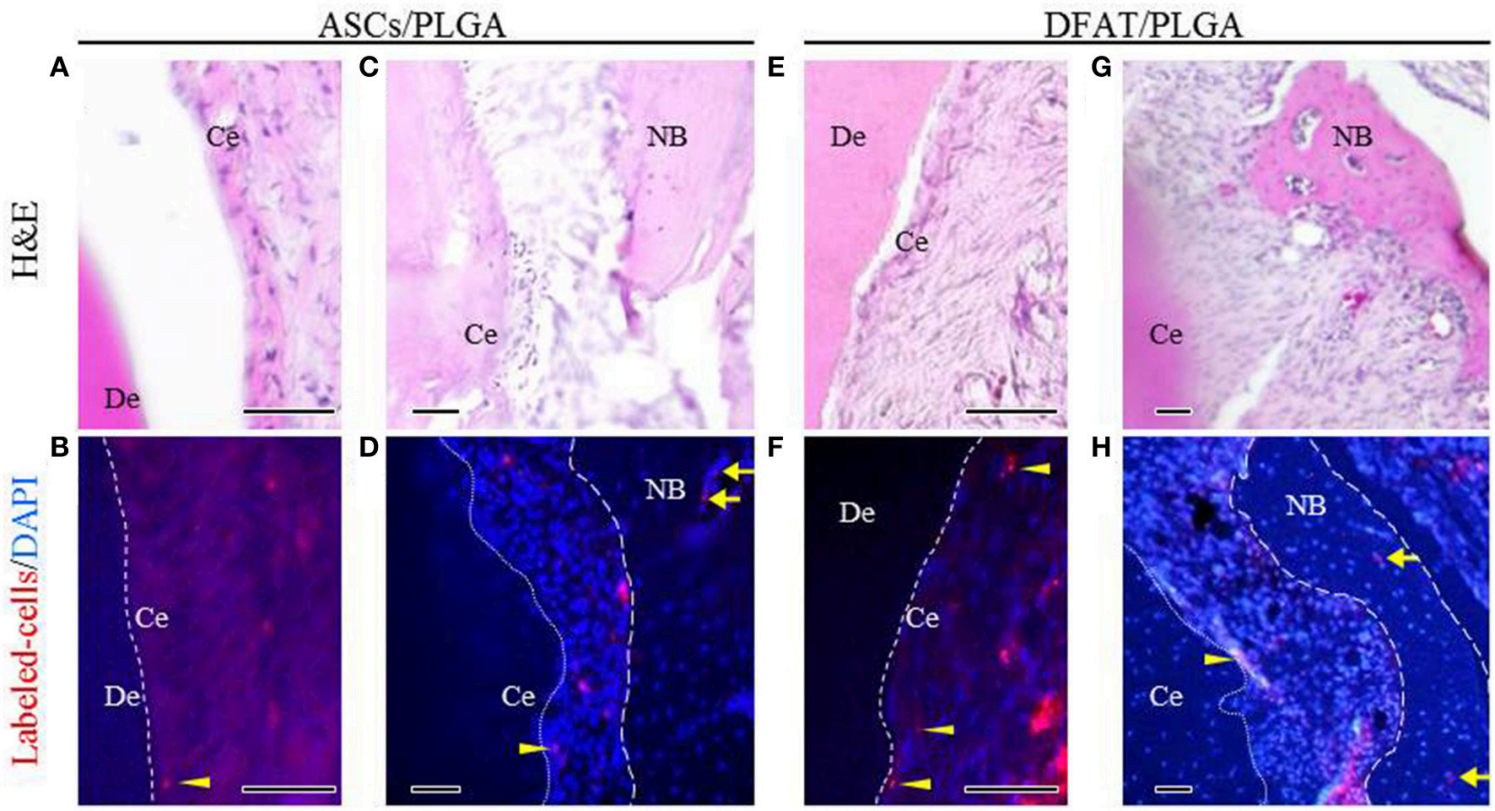
**Localization of fluorescent-labeled ASCs and DFAT cells analyzed via fluorescence microscopy 5 weeks after transplantation. (A,C,E,G)** Highly magnified fluorescence images of the fenestration defect in the distal root of the first molar. (H&E staining; scale bar: 50 μm). **(B,D,F,H)** The dotted line indicates newly formed periodontal ligament tissue between cementum and bone. Fluorescent-labeled ASCs and DFAT cells were observed in periodontal ligament beside the bone (arrows) and cementum (arrowheads). (Blue color: DAPI; Scale bar: 50 μm.). NB, newly formed bone; Ce, newly formed cementum; De, root dentin.

Fluorescent-labeled DFAT cells and ASCs were visualized in the newly formed alveolar bone and cementum, as well as in the newly formed periodontal tissues (Figures 10A–H).

## Discussion

The aim of this study was to assess the periodontal regenerative potential of DFAT cells derived from rat mature adipocytes in combination with solid PLGA scaffolds transplanted into surgically created periodontal tissue defects (King et al., 1997; Akita et al., 2014). In addition, we compared the proliferation and differentiation potential *in vitro* and periodontal tissue-forming capability *in vivo* of DFAT cells and ASCs.

A successful periodontal tissue-engineering strategy requires a suitable scaffold that maintains space for cell growth and differentiation. A suitable scaffold for periodontal tissue regeneration should be biodegradable, so that it can eventually be replaced by regenerated tissue (Rungsiyanont et al., 2012). We previously demonstrated the *in vivo* performance of a PLGA scaffold seeded with ASCs (Akita et al., 2014). When a PLAG-alone scaffold was transplanted into a periodontal defect in the present study, the defect space was occupied by connective tissue. In addition, newly formed connective tissue was observed around the remaining scaffold 5 weeks after transplantation and no inflammation was observed during the 5-week experimental period. These results suggest that the PLGA scaffold contribute to maintaining space for tissue regeneration in the periodontal defect because the structural integrity of the PLGA scaffold is higher than that of soft scaffolds such as those made from hydro gels and collagen sponge, which have been used in many other studies (Doğan et al., 2003; Kawaguchi et al., 2004; Zhao et al., 2004; Tobita et al., 2008; Li et al., 2009; Nuñez et al., 2012).

Interestingly, based on ALP activity and mineralization activity *in vitro*, DFAT cells showed higher osteoblastic differentiation capacity than ASCs. Furthermore, DFAT cells showed greater potential for hard tissue formation *in vivo* than ASCs by micro-CT analysis. These results indicate that the osteogenic potential of DFAT cells is higher than that of ASCs, which is consistent with previous findings (Kishimoto et al., 2013). In addition, based on Oil red O and Alcian blue analysis, DFAT cells and ASCs had similar adipogenic and chondrogenic differentiation potential. Our group has studied DFAT cells in term of tissue-specific functions and genes that regulate of differentiation, such as those encoding SFRP2, PRRX1, HEY2, AEBP1, PEG10, PRRX2, RUNX1, FZD7, and IGFBP5. The results indicate DFAT cells have a multilineage differentiation capacity and a feature of progenitor cells committed to various cell lineages rather than the stem cell fate (Ono et al., 2011). Furthermore, our previous study showed that when a single cell-derived clonal population of DFAT cells expressed the specific genes of various cell lineages, it was capable of differentiating into multiple lineages (Kazama et al., 2008; Matsumoto et al., 2008). Taken together, these findings suggest that DFAT cells could have the ability to differentiate into cementoblasts, fibroblasts, and osteoblasts.

In our study, we observed *in vivo* periodontal tissue regeneration including cementum, periodontal ligament, and alveolar bone following both transplantation of DFAT cells and transplantation of ASCs. For the functional repair of the cementum-ligament-bone complex, fibrous connective tissues must insert into the cementum, and bone to achieve optimal biomechanical integration. In our study, the PLGA-alone group showed no cementum or alveolar bone formation, This is because the damaged tissue does not naturally repair owing to the lack of MSCs in periodontal tissues. In contrast, when DFAT cells or ASCs were transplanted into the periodontal tissue defect, newly formed cementum, and alveolar bone formation was observed in the periodontal fenestration defect. Interestingly, the regenerated cementum and bone volume in the DFAT/PLGA group was significant higher than in the ASCs/PLGA group. This is the first comparative study on periodontal tissue regeneration to show that rat DFAT cells have highly potential for cementum and alveolar bone formation than ASCs. However, the rat is not a reliable animal model because rats have a very high capability of regenerating bone. Therefore, we need to examine the periodontal tissue regeneration potential of DFAT cells in a large animal model before clinical use is attempted.

In contrast to our methods, Sugawara, and Sato used DFAT cells that were cultured in an osteogenic differentiation induction medium before transplantation. They observed a small amount of a cementum on the exposed root surface (Sugawara and Sato, 2014). In our study, we found thicker cementum and alveolar bone formation. The discrepancy could be because of difference in the number of transplanted cells, the scaffold, and the treatment before transplantation. We seeded 1 × 10^6^ DFAT cells in the PLGA scaffold. In addition, DFAT cells in our study had not been induced to undergo differentiation into specialized cells *in vitro*. Further research is needed to clarify suitable cell number and suitable pre-transplantation treatment.

We specifically observed fibrous connective tissue in the newly formed cementum and alveolar bone in the DFAT cell group and the ASC group by using Picrosirius-red staining and Azan staining. Interestingly, the periodontal ligament space in the DFAT/PLGA was wider than that in the ASCs/PLGA. The results suggest that the transplanted DFAT cells participated in the repair of periodontal tissue via secretion of trophic factors or that they could directly participate into the various cell types constituting the periodontium (Chen et al., 2008; Gnecchi et al., 2008; Mooney and Vandenburgh, 2008; Chen and Jin, 2010). Our group has reported that DFAT cells secrete several cytokines associated with bone formation and angiogenesis (Kikuta et al., 2013). In addition, some GFP-positive DFAT cells clearly remained in the regenerated periodontal tissue area, as also reported in a previous study on periodontal tissue regeneration (Sugawara and Sato, 2014). Our analysis also revealed that the transplanted DFAT cells were detected at the injection site 5 weeks after transplantation. Both of these results suggest that at least some of the DFAT cells engrafted onto injected periodontal tissue and contributed to local periodontal tissue regeneration. Further research is required to determine specifically whether the labeled DFAT cells generated the differentiated cementoblasts, osteoblasts, and periodontal ligament fibroblasts. Previous studies have demonstrated that MSCs, at least in part, can differentiated into cementoblasts, osteoblasts, and fibroblasts in regenerated periodontal tissue (Hasegawa et al., 2006; Tobita et al., 2008).

In conclusion, we found a significantly greater increase in crucial tissues, cementum, and alveolar bone in the DFAT/PLGA group compared with the ASCs/PLGA group. The regenerated periodontium restored the architecture to an arrangement that was similar to the original architecture, collagen fibers inserted into the cementum, and alveolar bone layers. DFAT cells could be a promising cell source for periodontal tissue regeneration. However, the impact of the local differentiation microenvironment on DFAT cells and the mechanisms controlling the differentiation into cementoblasts, periodontal ligament fibroblasts, and osteoblasts remain to be examined further.

## Author contributions

DA: Performed most of work, in particular *in-vivo* study. KK: Developed the technique to isolate DFAT cells. YS: Supported the experiment performed by DA. T. Mashimo: Performed *in vivo* experiments. N. Tsurumachi: Isolated DFAT cells from rat. KY: Developed the scaffolds in our study. TK: Developed the scaffold. TT: Performed Cell culture and differentiation assay. N. Tsukimura: Made the experimental design. T. Matsumoto: Established the differentiation assay and cell culture technique. TI: Prepared the manuscript. KI: Managed the overall experimental design. MH: Controlled the overall paper until completion.

## Funding

This work was supported in part by Grant-in-Aid for Scientific Research (B and Houga) (21390528, 15H05037, and 15K15724 to MH, and 15H04607 to KK), research grants from the Promotion Project of Medical Clustering of Okinawa Prefecture (KK), Grants from the Dental Research Center, the Nihon University School of Dentistry (MH), Nihon University Multidisciplinary Research Grant for 2012 and 2013 (MH), and the Nihon University Graduate School of Dentistry research fund (10010105 to DA). This work was also supported by the MEXT-Supported Program for the Strategic Research Foundation at Private Universities (S1411018).

### Conflict of interest statement

The authors declare that the research was conducted in the absence of any commercial or financial relationships that could be construed as a potential conflict of interest.
